# GAP: From Sound Design to Practical Implementation in Clinical Trials for Traditional Chinese Medicine

**DOI:** 10.1155/2014/560838

**Published:** 2014-02-25

**Authors:** Hongcai Shang, Boli Zhang, Zhaoxiang Bian, Youping Li, Mike Clarke, Nicola Robinson

**Affiliations:** ^1^Tianjin University of Traditional Chinese Medicine, 88 Yuquan Road, Tianjin 300193, China; ^2^China Academy of Chinese Medical Sciences, 16 Nanxiao Street, Beijing 100700, China; ^3^School of Chinese Medicine, Hong Kong Baptist University, 7 Baptist University Road, Kowloon Tong, Hong Kong; ^4^Chinese Cochrane/Evidence-Based Medicine Centre, West China Hospital, Sichuan University, 37 Guoxue Lane, Chengdu 610041, China; ^5^All-Ireland Hub for Trials Methodology Research, Queen's University, Belfast BT7 1NN, UK; ^6^London South Bank University, 103 Borough Road, London SE10AA, UK

The past few years have witnessed encouraging progress in improving the methodological quality of clinical research of traditional Chinese medicine (TCM). This improvement has contributed to wider academic acceptance of the findings of TCM clinical studies, which were previously deemed dubious. As a proof of this statement, one clinical study testing the effects of a Chinese patent drug Qili Qiangxin Capsules on chronic heart failure has just published a research article on the Journal of the American College of Cardiology, a medical journal of international prestige. However, a sound and scientific design does not always see to its practicality in the conduct of the study, and in fact we observed a widening gap between the two elements. In this special issue, we called for papers discussing efforts to bridge the gap between scientific design and practical implementation of clinical research with TCM.

In Y. Xing et al.'s review article, “ *The effects of Wenxin Keli on P-wave dispersion and maintenance of sinus rhythm in patients with paroxysmal atrial fibrillation: a meta-analysis of randomized controlled trials*,” they synthesized and assessed the results of randomized controlled trials (RCTs) in order to address a specific clinical question and critically commented on several practical issues in the conduct of TCM clinical research.

In another review article, “*Clinical research of traditional Chinese medicine needs to develop its own system of core outcome sets*,” L. Zhang et al. proposed constructing a system of core outcome sets that caters for clinical evaluation of TCM after reviewing existing problems in the choice of outcomes to be assessed in a clinical study.

Patient value is one of the three components necessary for evidence-based clinical decision-making. Patient values and its various manifestations also have a crucial role to play in the conduct of a clinical research and in the evaluation of TCM efficacy and safety. W. Mu and H. Shang called for academic attention to this area of research in their paper entitled “*Understanding patient values and the manifestations in clinical research with traditional Chinese medicine—with practical suggestions for trial design and implementation*.”

Issues around compliance control in clinical research of TCM always attract interests. W. Zheng et al. summarized practical barriers to the management of incompliant behaviors from past experiences of being a clinical trial investigator and put up with point-to-point solutions in their review article entitled “*Improving participant adherence in clinical research of traditional Chinese medicine*.”

In H. Yu et al.'s research article “*Clinical study on the prevention of oxaliplatin-induced neurotoxicity with Guilongtongluofang: results of a randomized, double-blind, placebo-controlled Trial*,” they introduced the measures they took to balance the rigorousness of study design and its practicality and provided a successful example.

In a similar vein, J. Pu et al. explained in the research article “*Chinese medicine Shensongyangxin is effective for patients with bradycardia: results of a randomized, double-blind, placebo-controlled multicenter trial*” their experiences of taking consideration of real clinical circumstances into the process of trial design, so as to narrow the gap between research design and implementation.

*Hongcai Shang* *Hongcai Shang* *Boli Zhang* *Boli Zhang* *Zhaoxiang Bian* *Zhaoxiang Bian* *Youping Li* *Youping Li* *Mike Clarke* *Mike Clarke* *Nicola Robinson* *Nicola Robinson*

